# The Influence of the Family on Adolescent Sexual Experience: A Comparison between Baltimore and Johannesburg

**DOI:** 10.1371/journal.pone.0166032

**Published:** 2016-11-07

**Authors:** Kristin Mmari, Amanda M. Kalamar, Heena Brahmbhatt, Emilie Venables

**Affiliations:** 1 Johns Hopkins Bloomberg School of Public Health, Department of Population, Family and Reproductive Health, Baltimore, Maryland, United States of America; 2 Division of Social and Behavioural Sciences, School of Public Health and Family Medicine, University of Cape Town, Cape Town, South Africa; London School of Hygiene and Tropical Medicine, UNITED KINGDOM

## Abstract

The main objective of this paper is to understand the role of the family on the sexual experiences of adolescents from urban, disadvantaged settings in Baltimore and Johannesburg. Data were collected as part of the WAVE study, a global study of disadvantaged youth in five cities. Qualitative data were based on key informant interviews, a Photovoice exercise, community mapping, focus groups and in-depth interviews with adolescents. Quantitative data were gathered from an ACASI survey that was administered to approximately 450–500 adolescents per site. Results from the qualitative data revealed that while parents were viewed as important sources of information for sexual and reproductive health, they were often not present in the adolescents’ lives. This lack of parental presence was perceived to result in adolescents feeling an overall lack of adult support and guidance. The impact of parental presence and support on adolescent sexual experience was further examined from the quantitative data and revealed a complex picture. In both Baltimore and Johannesburg, female adolescents who were raised by other relatives were *less* likely to report having had sex compared to those raised by two biological parents, which was not observed for males. In Johannesburg, female adolescents who were paternal orphans were less likely to have had sex compared to non-orphans; the opposite was true among males. Finally, in both sites, female adolescents who had been exposed to violence were more likely to have had sex compared to those who had not; for males, there was no significant relationship. The study demonstrates the powerful influence of both context and gender for understanding the influences of the family on adolescent sexual behaviors. Programs aiming to reduce adolescent sexual risk behaviors the need to understand the complex influences on risk behaviors in different settings and in particular, the role of mothers and fathers. Prevention strategies need to also understand and incorporate gender-specific messages and interventions in order to address the high risk of sexual behaviors among adolescents in these settings.

## Introduction

Over the past two decades there has been a large body of research substantiating the powerful influence of the family on adolescent sexual health behaviors and outcomes [1–13]. In general, studies found that adolescents in married, biological two-parent families are less likely to engage in unprotected sex and early sexual initiation compared to adolescents from single parent, cohabiting stepfather, and married stepfather families [2]. In fact, irrespective of whether it is a low, middle or high-income country, adolescents raised in single parent households have an increased probability of both early sexual debut and pregnancy [3–5]. While family structure has been suggested to have an indirect effect on adolescent sexual behavior, parent-child interactions and processes are believed to have a more direct effect on such behaviors. For instance, closer parent-adolescent relationships and greater parental support and monitoring have been shown across multiple contexts to reduce adolescents’ engagement in sexual risk behaviors [6–10].

Additionally, greater parent-child communication (especially related to sexual and reproductive health) has been associated with greater protection and less sexual risk [11–13]. Yet, depending on the context, the influence of parent-child communication on adolescent sexual health outcomes varies. For example, while a study conducted among four African countries found that parent-child communication about sexual activity was associated with delayed sexual debut among female adolescents [11], studies in other settings have found the opposite to be true, especially among males [12–13].

A major limitation of previous research, however, is that studies often failed to consider the particular context such as family structure, within which sexual behaviors were occurring. In the *Well-being of Adolescents in Vulnerable Environments* (WAVE) study—adolescents from two of the five sites that participated, Baltimore and Johannesburg, not only exhibited similar levels of sexual activity and outcomes but also similar types of family structure. Specifically, as distinct from Ibadan (Nigeria), Shanghai (China), and New Delhi (India), adolescents from Baltimore and Johannesburg were both the most sexually experienced and most likely to be raised either in single-parent or other relative/non-relative households [14]. In Baltimore, about 28% of adolescents lived with one parent and an additional 24% were raised by other relatives or non-relatives. In Johannesburg, while less than 10 percent (9.7%) were raised by one parent, more than a third of the sample (36%) were raised by other relatives or non-relatives (see Table 1 below). Among those living with other relatives or non-relatives, 30% were paternal orphans, almost eight percent were maternal orphans, and nearly six percent were double orphans. Given the striking similarities in both sexual risk taking and family structure among adolescents in Baltimore and Johannesburg, further analyses were warranted to explore commonalities between the family environment and sexual experience among adolescents in these two sites.

**Table 1:** Selected Characteristics of Survey Sample; W% = weighted percent.

	Baltimore, W% (N)	Johannesburg, W% (N)
**N**	456	496
**Sociodemographic Characteristics**		
**Sex**		
Male	46.4 (263)	56.7 (272)
Female	53.6 (193)	43.3 (224)
**Age group**	43.0 (252)57.0 (204)16.8	50.6 (123)49.4 (373)16.6
15–16	43.0 (252)	50.6 (123)
17–19	57.0 (204)	49.4 (373)
Mean age (SE)	16.8	16.6
**School enrollment**		
Enrolled	81.0 (388)	84.3 (406)
Not enrolled	19.0 (67)	15.7 (88)
Among not enrolled, % who graduate	66.6 (47)	42.9 (32)
**Perceived wealth status**		
Better than most	36.7 (157)	20.3 (110)
Same as most	54.2 (264)	68.5 (342)
Worse than most	7.1 (30)	11.2 (45)
**Raised by**		
Two parents, biological	37.9 (154)	33.9 (167)
Two parents, one non-biological	12.7 (54)	18.8 (104)
One parent	24.4 (109)	9.6 (48)
Other relatives	21.5 (129)	36.4 (170)
Other non-relatives	3.2 (18)	1.3 (6)
**Female Adult Support at Home**	10.0 (3.2)	9.1 (3.3)
Mean (SD)		
**Male Adult Support at Home**	7.4 (4.0)	7.3 (3.5)
Mean (SD)		
**Sexual and Violence Outcomes**		
**Ever had sexual intercourse**	79.0 (359)	70.0 (346)
**Experienced home violence**	20.4 (106)	42.6 (205)

In the United States, while the influence of family structure and stability on adolescent health behaviors has been studied comparing whites and blacks [2,15]—with family structure having a far greater effect on early sexual debut among white compared to black adolescents, very little work has examined these associations cross-nationally. In the WAVE study, while the adolescent samples for both the qualitative and quantitative components of this study were overwhelmingly black and from economically disadvantaged neighborhoods in both Baltimore and Johannesburg, the cultural context that surrounds family structure and sexual activity are quite different. In Baltimore, family instability is caused largely by parental substance use and incarceration, while in Johannesburg it is primarily due to parental death, assumed mainly from AIDS and other infectious diseases such as tuberculosis and labor migration (primarily of the father). While it is reasonable to believe that the commonalities of poverty and lack of two biological parents in both sites explain early sexual activity, this has yet to be tested. Further, if it is the case, the processes by which those influences are exerted (e.g., lack of parental control and presence leading to a sense of abandonment and detachment from their primary caregiver) have yet to be delineated, especially across urban contexts where the underlying determinants of family structure and stability are so distinct from each other. Additionally, given that qualitative data gathered from the WAVE study also addressed perceptions from key informants about how the family influenced the health of adolescents, analyzing this data may also provide additional insight about the family’s influence on adolescent sexual activity. Using both the qualitative and quantitative data from the WAVE study, the primary objectives of this paper are: 1) to explore the factors within the family environment that are perceived to influence the sexual experience among adolescents in both Baltimore and Johannesburg, and 2) to examine how the associations between family structure, support, and sexual experience differ between the two urban sites.

## Methods

### Research Design

The WAVE study is the first global study to focus on very disadvantaged adolescents (aged 15–19 years) from urban environments in five cities: Baltimore (USA), Ibadan (Nigeria), Johannesburg (South Africa), New Delhi (India), and Shanghai (China). The first and formative phase of the study was qualitative, and was launched in June 2011 to explore adolescents’ perceptions of health and health challenges in their environments and to describe the factors within their communities that are perceived to influence their health seeking behaviors. Data were collected using identical research protocols across the five study sites: key informant interviews among representatives from schools, faith-, community- and youth-serving organizations; in-depth interviews among adolescents; community mapping and focus groups among adolescents; and a Photovoice exercise among adolescents [16].

For phase 2, a survey using an audio computer assisted survey instrument (ACASI) was conducted between March and August of 2013 among adolescents in the same five urban sites. To recruit adolescents for the survey, each site used a respondent-driven sampling (RDS) methodology, which consisted of selecting adolescents as “seeds” to serve as the initial contacts for recruitment [17].

### Ethics Statement

Consent procedures were standardized across sites. Written consent was obtained from adolescent participants aged 18 years and over. For adolescents younger than 18 years, a combined written parental/guardian consent and child assent form was signed. A parent or guardian could include anyone who had legal authority over the child, which in some cases meant directors of homeless shelters or, in Baltimore, the Social Services Administration for foster children. All research protocols were approved by the Johns Hopkins Bloomberg School of Public Health IRB, and the Human Research Ethics Committee of the University of the Witwatersrand in South Africa.

### Survey Measures

#### Family Structure

For the present analyses, we used two categorical measures of family structure. The first measure asked adolescents about the individual (s) who raised them, with the reference category being two biological parents. Other family constellations included: one biological and one step or adoptive parent, two step or adoptive parents, one parent whether biologic or not, other relative (e.g., grandparent) family and non-relative family. In Johannesburg, in addition to the family structure we include a measure of orphan status to account for the impact of the HIV epidemic on family structure. While the reference category of being raised by two biological parents remained the same, for those raised in other family constellations, orphan status was captured and defined as paternal orphan, a maternal orphan, and double orphan.

#### Adult Support at Home

To assess caregiver support from an adult in the home, we use two measures each consisting of four items with four response options (‘never true’ (0) to ‘always true’ (4)), for a total range of 0–12. Both measures were adapted from a multi-site adolescent health survey in the Caribbean [18]. The four items, asked identically for both male and female adult support, asked whether there was a male/female adult presently in the respondent’s home who 1) “expects you to follow the rules”; 2) “believes that you will be successful”; 3) “you can turn to when you have a problem”; and 4) “listens to you when you have something to say”. The first scale measured the extent of support from a male adult at home (alpha 0.88 in Baltimore and 0.80 in Johannesburg) and the second measured the extent of support from a female adult at home (alpha 0.92 in Baltimore and 0.84 in Johannesburg).

#### Home Violence Victimization

To capture home violence exposure, we created a binary variable (yes/no) from a set of five questions that asked respondents about different instances of victimization experienced directly by the adolescent from any member of their household, such as being pushed or grabbed, threatened with a weapon, or assaulted with a weapon.

### Data Analysis

#### Qualitative data

For the purposes of the present analyses, only the transcripts from Baltimore and Johannesburg were examined to gain a more comprehensive understanding of family influence on adolescent sexual behavior. This included analyzing transcripts from eight Photovoice discussions (four in each city), 34 key informant interviews (16 in Baltimore and 18 in Johannesburg), 34 in-depth interviews among youth (17 in both cities), and 14 community-mapping exercises and focus groups (five in Baltimore and eight in Johannesburg). Matrices of the codes that related to family support, the influence of family on adolescent health, and perceived factors that influence sexual behaviors were developed to examine similarities and differences among participant groups and methods.

#### Quantitative data

Survey data were weighted to account for the RDS design of the study and weights were generated via the RDS-II estimator to account for inter-cluster correlation, network size, and non-independence of observations [19]. Weighted logistic regressions were used to explore the relationship between parenting factors and ever having sex among male and female adolescents. The fully adjusted model includes the parenting variables as well as age, school enrollment status, housing stability, and in Johannesburg whether the respondent was foreign born.

## Results

Results are presented first by the qualitative findings, followed by the multivariate analysis.

### Family Factors that Influence Sexual Activity and Risk Taking

#### Lack of Parental Support

For male and female adolescents and key informants living in both Baltimore and Johannesburg, there was a serious concern about the lack of parental guidance or support. In Baltimore, key informants explained that parents even when physically present were often not truly available as caregivers for their children. Both adolescents and key informants frequently noted that parents were often involved with substance use, including alcohol, marijuana, and crack. Likewise, fathers too often were incarcerated or otherwise absent.

A lot of parents is out here on drugs, honestly, honestly. (IDI, Baltimore adolescent girl)

I think the biggest and most heartbreaking challenge that these kids face is the lack of family, parenting. There's no one at home that is an appropriate caregiver—that is a healthy caregiver. (Key Informant Interview, Baltimore)

Young adolescents were often left to raise themselves and their younger siblings, even if they were living with their parents. The lack of emotional support for these adolescents was apparent among informants from Baltimore.

I just know our kids are starved. They're absolutely starved. Attention starved, attention deprived. They just really want somebody to just love on them, show them they care about them, whatever that looks like. (Key Informant Interview, Baltimore)

An absence of parents as caregivers was also frequently noted among Johannesburg participants, though the circumstances were different from those in Baltimore. Specifically, stories told in Johannesburg revealed the huge impact of the HIV/AIDS epidemic on the social environment; additionally, many stories discussed fathers living away from them either because of relationship dissolution or work. The perception of being alone was observed among both male and female adolescents.

I do everything on my own. Even at home, I do everything on my own…I am one of those people that keep problems to myself and then somehow these problems will go away. (IDI, Johannesburg adolescent boy)

Many key informants and adolescents described the increased vulnerability of orphaned children who grow up in single-parent households and the impact it can have on their lives.

We find that most of them are coming from single headed families where there is only one parent and the other party is not there. So they are very vulnerable. (Key Informant Interview, Johannesburg)

#### Parental Communication

Another common theme that was mentioned by participants in both cities was the absence of sexual education and the lack of communication between parents/guardians and their adolescent children about sex. In Baltimore, young women in particular, often reported it was rare for a parent/guardian to discuss sexual and reproductive health issues with them. Notably, many of the adolescent study participants reported that they wished they could receive information to understand the emotional and physical consequences of sexual initiation or ways to protect themselves from sexually transmitted infections or pregnancy. Instead, as mentioned by several key informants, adolescents were unable to recognize important aspects of healthy sexual relationships and advocate for themselves during discussions with their partners.

A lot of STDs… because a lot of unprotected sex. That stems from also people not being able to learn from their parents how to protect themselves and then not going to school to go to health class. Everything leads back to that so there's a lot of STDs, a lot of babies, a lot of sicknesses, a lot of unhealthiness because we're not taking care of ourselves because we haven't learned. (IDI, Baltimore adolescent girl)

Key informants further expressed concern that this lack of parental communication and guidance about sexual health impeded female adolescents’ ability to make healthy decisions about their sexuality.

I know this one girl, she's a good little girl and does good in school and all that.

But her mother not telling her nothing about sex. She's asking about sex and I think she's about to be 16. She asked her mother about sex, and her mother was like "no, I'm not telling you because you be getting smart.” (Key Informant Interview, Baltimore)

Participants in Johannesburg also noted a breakdown of parent/adolescent communication as an issue of high concern. It seemed that, even in circumstances where a young adolescent was living with or receiving some support from one of her parents, this support did not extend to a meaningful discussion of healthy sexual behavior. In-depth interviews revealed that young women were generally concerned about what their caregivers—be it a single parent, aunt, or grandparent—would think of them if they became pregnant. Although they were warned not to get pregnant or not to have a boyfriend, and to instead focus on their education, they were not provided with information about healthy relationships and safe sexual practices despite the impact HIV has on many of their everyday lives.

You see the major [problem] is broken families. Before you even start talking about habits, the home itself it’s like not a good structure for the family. It’s either this one lives with a single father or single mother or lives with granny so this family bond is not there. It’s one of the major challenges so when they meet others they don’t know how to behave as a family and then we come onto things like teenagers taking dating and sex as one of their first priorities. So here and there you have teenagers pregnancies. (Key Informant Interview, Johannesburg)

#### Domestic and Intimate Partner Violence

As already illustrated from the quantitative data in Table 1, domestic violence was common in the lives of young adolescents, and particularly among females in both settings. In Baltimore, interviews revealed that female adolescents were vulnerable to multiple forms of violence, from both their families and their partners. Several participants noted that starting from when they were young children they would observe violent relationships between parents or other adults. Several female adolescents in Baltimore noted that violence and disrespect comes to be viewed as routine parts of relationships and perhaps even an acceptable or expected behavior.

So, our young ladies learn abuse at a very early age. Not only do they have it in the home, and that's all they knew, a lot of them. Whether it be, again, verbal, mentally, physically, whatever the case may be. So, that's their identity or that's their definition of love. (Key Informant Interview, Baltimore)

One key informant in Baltimore explained that because females are more likely to report abuse than males, most of her knowledge about domestic abuse came from her female clients. Males, in contrast, were more likely to keep it to themselves. Among her female clients, a large proportion were dealing with mother/daughter relational problems, which had caused significant emotional damage from one generation to the next.

The trends that I have seen is the mother, and her mother. It’s generational. It’s just passed down. It’s a cycle of abuse, a cycle of violence, and a cycle of substance abuse.

Key informants and adolescents in Johannesburg shared remarkably similar information about relationship violence—in the form of violence against their mothers, female care-givers and sisters—being commonly witnessed by adolescents, and reiterated that such violence comes to be viewed as an acceptable norm. Young adolescents learned that abuse was likely within multiple facets of their life and that the communities in which they live have not and will not come together to address or challenge it. Even if young people in Johannesburg–male and female–did not always talk directly about violence they had experienced themselves, it was apparent that violence–especially against women—was an everyday part of life in their inner-city environment.

Challenges are harder for girls. There’s a lot of rape that happens and most of the crimes done are to women. Men are more likely to fight back or run away so they can’t be abused. We are being pushed and hurt more than the men in this area. A lot. (IDI, female adolescent, Johannesburg)

It is because if you look at their background, they've been abused from their families, they've been abused on the street, you know, so it's a circle of abuse. (Key Informant Interview, Johannesburg)

### Multivariate Analysis of Associations Between Family Contextual Factors and Sexual Experience

Table 2 illustrates the associations between family contextual factors and ever had sex, stratified by gender. Notably, there were not only large gender differences within each site, but there were also differences between the sites. In Baltimore, male adolescents who were raised by non-relatives were more than three times as likely to have ever had sex compared to those raised by two biological parents (p<0.01). Interestingly, among female adolescents in Baltimore, being raised by other relatives was a protective factor. These female adolescents were *less* likely to have had sex compared to those raised by both parents (p<0.05). In Johannesburg, while male adolescents’ sexual experience did not seem to be influenced at all by family structure, female adolescents’ sexual experience was–but not in the expected direction. Notably, female adolescents who were raised in two-parent (where there was at least one adoptive or step parent), other relative or non-relative households, were actually less likely to have sex compared to female adolescents who were raised in two-parent (both biological) households.

**Table 2:** Ever Had Sex: Logistic Regression Results.

	Baltimore				Johannesburg			
	Boys (N = 248)		Girls (N = 186)		Boys (N = 249)		Girls (N = 214)	
	AOR	95% CI	AOR	95% CI	AOR	CI	AOR	CI
**Family Structure**								
Two Parents (biological) (Ref)								
Two Parents (One/Both Step/Adoptive)	2.32	(0.94, 5.72)	1.66	(0.55, 5.02)	0.75	(0.23, 2.50)	0.28***	(0.21, 0.38)
One Parent	0.47	(0.19, 1.15)	1.03	(0.59, 1.80)	1.07	(0.04, 27.32)	1.16	(0.46, 2.96)
Other Relatives	1.01	(0.56, 1.87)	0.34*	(0.16, 0.74)	0.98	(0.35, 2.72)	0.43*	(0.20, 0.96)
Other Non-Relatives	3.51**	(1.57, 7.86)	0.27	(0.02, 3.04)	Cell size too small	-	0.42***	(0.30, 0.59)
**Orphan Status (Johannesburg only)**								
Not an orphan (Ref)								
Lost only father	-	-	-	-	2.40*	(1.13, 5.11)	0.58**	(0.42, 0.79)
Lost only mother	-	-	-	-	0.50	(0.06, 3.81)	4.96***	(2.71, 9.08)
Lost both parents	-	-	-	-	1.83	(0.53, 6.41)	4.81**	(1.62, 14.27)
**Adult Support at Home**								
Adult Male Support at Home	0.96	(0.92, 1.00)	1.13**	(1.06, 1.21)	0.98	(0.89, 1.09)	1.04	(0.90, 1.19)
Adult Female Support at Home	1.06	(0.82, 1.37)	0.63***	(0.52, 0.77)	1.07	(0.91, 1.24)	1.01	(0.89, 1.15)
**Home Violence Victimization**								
No (Ref)								
Yes	0.86	(0.82, 1.37)	2.65***	(1.93, 3.63)	1.94	(0.82, 4.57)	2.34**	(1.34, 4.10)

* p<0..05

** p<0.01

*** p<0.001

All analyses are weighted for complex survey design and adjusted for age, school enrollment, unstably housed, and foreign born (Johannesburg only)

Given that a fairly large percentage of adolescents in both Baltimore and Johannesburg experience violence in the home (20% in Baltimore, more than 40% in Johannesburg), we also wanted to know whether violence was more common among female adolescents living in a two-biological parent household versus the other types of family structure. Indeed, for female adolescents in Johannesburg, this was precisely the case. In fact, bivariate associations found that female adolescents living in two-biological parent households were more likely to experience violence in the home compared to any other family structures, though the statistical significance was mixed (results available from the corresponding author). In Baltimore, however, female adolescents living in two-biological parent households were *less* likely to experience violence in the home compared to other family structures, with the exception of those living in two-parent, non-biological parent households, though again these unadjusted associations have mixed statistical significance.

Examining the association between ever had sex and orphan status among adolescents in Johannesburg, we saw a somewhat similar picture as we did with family structure. Among male adolescents, paternal orphans were more than twice as likely to have ever had sex compared to those who were raised by both biological parents (p<0.05). Among the female adolescents in Johannesburg, an opposite association was observed. Female adolescents who were paternal orphans were actually significantly *less* likely to have ever had sex compared to those raised by both biological parents (p<0.01). On the other hand, female adolescents who were maternal orphans were nearly five times more likely to have had sex compared to those raised by both biological parents (p<0.001); and female adolescents who were double orphans were also nearly five times more likely to have had sex compared to those who were not orphaned (p<0.01). It is also important to point out here that there was little correlation between being raised by a single parent and orphan status (correlation = 0.35) among adolescents in Johannesburg, which indicates that both family structure and orphan status were independently influencing sexual behavior.

An interesting relationship also emerged with male and female adult support at home and adolescent sexual experience–but was only significant among female adolescents in Baltimore. Female adolescents who received adult male support at home were significantly more likely to have had sex compared to those who didn’t receive this support (p<0.01). An opposite relationship, however, was found with female adult support at home. Female adolescents who received female adult support at home were significantly less likely to have had sex compared to those who didn’t receive this support (p<0.01).

The association between experiencing violence at home and ever having had sex appeared to also be strongly influenced by gender. In both Baltimore and Johannesburg, there was no significant relationship between experiencing violence at home and ever having sex among male adolescents. For female adolescents, however, those who were victims of home violence were more than twice as likely to have ever had sex compared to those who were not victims (p<0.001 in Baltimore and p<0.01 in Johannesburg).

## Discussion

The main objective of this paper was to examine the association between the family environment and adolescent sexual experience among adolescents living in Baltimore and Johannesburg. Although the environments, cultures and reasons for parental absence were significantly different in the two cities, the consequences for adolescent sexual and reproductive health were comparable. Qualitative data revealed that while parents were viewed as important sources of information for sexual and reproductive health, they were often not present in adolescents’ lives. This lack of parental presence was perceived to result in adolescents feeling an overall lack of adult support and guidance. Although the influences on adolescent risk behavior are complex and not fully understood, it was important to understand how this lack of support and guidance influenced adolescent sexual behaviors and if there were differences by gender and site. The multivariate analysis revealed that in both sites, overall, female adolescents’ sexual experience seemed to be more influenced by family structure than were male adolescents. However, contrary to most studies [2–4], being raised in a two-biological parent household was not necessarily the most protective. In Baltimore, for example, female adolescents who were raised by other relatives were actually less likely to be sexually experienced compared to those raised by two biological parents. Although we did not ask adolescents about the quality of their parents’ relationship, there have been studies that have shown that couples and families who face economic hardships also experience higher levels of relationship conflict–which has also been shown to be related to female adolescents’ sexual experiences [20–22]. In addition, as shown by the quotes shared by adolescents from Baltimore, many parents, even if physically present, were not available, and many adolescents described the prevalence of drug and alcohol use and violence in their homes. On the other hand, among male adolescents in Baltimore, one of the strongest risk factors for sexual behavior was being raised by non-relatives. In Johannesburg, only female adolescents’ sexual experience was influenced by family structure, and similar to females in Baltimore, those raised by two biological parents were more likely to be sexually experienced. With the exception of being raised by a single parent, female adolescents who were raised in any other type of family structure composition–whether it be an adoptive/step parent family, other relatives, or other non-relatives—were less likely to be sexually experienced compared to those raised in a two-parent biological household. Although factors that may explain this were not assessed in this study, given the high levels of violence, drug use, and instability that exist within the family environment, females may be particularly vulnerable even if they live in two-parent households.

The findings by orphan status in Johannesburg may offer further explanation to this notion. Female adolescents who had lost a mother or both parents were more likely to be sexually experienced compared to those who were not orphaned. However, if they had lost only a father, they were less likely to be sexually experienced compared to those who were not orphaned, and this is consistent with previous studies that have underscored the importance of surviving mothers on adolescent health and well-being [23–25]. While it is a different family factor, a somewhat similar pattern was observed for adult support in Baltimore. Female adolescents who received male support from the home were more likely to be sexually experienced, whereas female adolescents who received female support from the home were less likely. There was no association, however, for male adolescents.

Indeed, the role of gender in these relationships is especially important. According to a recent literature review which specifically examined gender as a moderator in the link between parenting and adolescent sexual behavior, adolescent female sexual behaviors are more influenced by the parent-child relationship than their male counterparts [26]. To explain this, the authors suggested that female adolescents (in general) place much more meaning on interpersonal relationships (such as the parent-child relationship) and therefore may be more affected by the ability of their parents to provide a supportive relationship. The role of gender is also important when we look at the influence of mother versus father. Studies in the United States, for example, have found that while mothers provide similar levels of parental support regardless of the family structure, stepfathers are less effective caretakers of their non-biological children; however, Simons and colleagues suggest that when mothers have help and support from a secondary caretaker, adolescent outcomes are more positive [27]. This may also help to explain why being a paternal orphan was protective against sexual debut among female adolescents in Johannesburg whereas being a maternal or double orphan increased the report of ever sex five-fold. In such a setting, the extended family still exerts a strong level of support; the fact that this was observed only among paternal orphans–when the biological mother is still the primary caretaker–suggests this supportive influence.

The other common finding observed from both the qualitative and the quantitative data in both sites was the influence of domestic violence on adolescent sexual experience. Again, this only affected female adolescents’ sexual experience. Indeed, research specific to low-income urban settings has shown that sexual violence may be common and that such abuse, when experienced earlier in childhood, may be associated with sexual risk behaviors later in life (and, specifically, risk behaviors related to HIV) [28–29]. The fact that female adolescents were more affected by domestic violence compared to male adolescents may reflect either that they might have been more exposed to the violence in the household, that they were potential victims of the violence themselves, or as discussed earlier, that female adolescents place more meaning on the family relationships and are therefore more affected by their breakdown. In Johannesburg, given that a higher proportion of female adolescents in two-biological parent households experienced violence in the home compared to those in other types of family structures, this may explain, at least partially, why these female adolescents were also more likely to be sexually experienced. In Baltimore, the higher likelihood of female adolescent sexual activity in two-biological parent households is likely related to other factors. Qualitative data, for example revealed that even though adolescents may be living with both parents, they are not receiving attention from them and therefore, may not be receiving a level of stability that is usually associated with two-parent households. Additionally, research conducted by Kapungu and colleagues found that among a sample of African American adolescents living in an impoverished urban area, female adolescents who were raised in high control/low warmth (authoritarian) households were at higher risk for engaging in earlier sexual activity; the opposite was observed for males [30]. Whether it is an authoritarian type of parenting or simply the lack of attention that is contributing towards sexual activity among female adolescents in Baltimore is still not clear; what is most interesting, however, is that for females at least, two-parent households may not necessarily be the most advantageous. Further research is needed to clarify these mechanisms.

*Study Limitations*: It’s important to point out several limitations with our study. First, data were collected from very specific neighborhoods within each of the WAVE cities. Though both communities are low-income areas of each city, our findings may not be representative of the experience of adolescents across similar neighborhoods even within the same cities. However, to address this challenge, the research teams in each site made sure that adolescent participants were recruited in a representative way, using a variety of recruitment methods, including passing out flyers in the neighborhood, and at community events, as well as using the key informants to help recruit adolescents. Teams also ensured that selected key informants were well-recognized community figures who would have reliable information and respected perspectives to share regarding the state of and barriers to adolescent health. Though the qualitative data in these analyses come from two specific communities, we are confident that the methodology employed and the precautions taken above add strength to the validity of our findings. Additional limitations may exist with our quantitative data. First, we did not ask any questions in the survey about parent-child communication, and specifically communication about sexual behaviors, and were therefore unable to assess its effect. Secondly, the sample for this study are adolescents 15–19 years old, an age when normative and healthy sexual development and experience is also occurring. While we only examined sexual experience (ever had sex), it’s important to point out that this should not be perceived necessarily as a sexual risk behavior. Finally, this is a cross-sectional study and as a result, we are unable to determine causality in any of the relationships observed.

## Conclusions

Despite the limitations, while this study’s primary objective was to examine and compare the influence of the family on adolescent sexual experience in Baltimore and Johannesburg, the findings emphasize the importance of both context and gender on the relationship between family and sexual experiences. The use of qualitative and quantitative data also provided context and explanations for some of the findings of our study regarding sexual behaviors and family structure. In both sites, female adolescents’ sexual experience was the most affected by factors within the family environment and, contrary to expectations, was greater among those living in two-parent households. While a lack of communication with parents on sexual health was highlighted in the in-depth interviews, there are potentially different mechanisms at play in the different settings. In Johannesburg, violence may have been a larger contributor to female sexual experience in two-parent households, while in Baltimore, other factors such as drug use are likely to contribute to the negative influence of “father” support and presence in such a setting. In urban poor settings where there are a high proportion of adolescents living in non-two parent households, this study also suggests that additional support should be targeted to mothers and other types of caregivers. At the same time, it is important to not assume that adolescents living in two-parent households are any better off than those living in other types of family structures–and for female adolescents in such households, this is especially relevant. Both parents need to therefore be educated about the vital role that they play–especially in their daughters’ sexual development and future health behaviors–and the parent-child relationship should be taken into account when designing and implementing programs for young people.

## Supporting Information

S1 Table Ever Had Sex: Logistic Regression Results from Two Models. (DOCX)
