# Supplementary material for: The Influence of the Family on Adolescent Sexual Experience: A Comparison between Baltimore and Johannesburg
Source: PLoS One. 2016 Nov 7;11(11):e0166032. doi: 10.1371/journal.pone.0166032 (PMC5098750; doi:10.1371/journal.pone.0166032)
Supplement: S1 Table — (DOCX) [file pone.0166032.s001.docx]

| **S1 Table. Ever Had Sex: Logistic Regression Results from Two Models** | | | | | | | | | | | | | |
| --- | --- | --- | --- | --- | --- | --- | --- | --- | --- | --- | --- | --- | --- |
|  | **Baltimore** | | | |  |  |  | **Johannesburg** | | | | | |
|  | Boys (N=248) | | Girls (N=186) | |  | Boys (N=249) | | Boys (N=249) | | Girls (N=214) | | Girls (N=214) | |
|  | AOR | 95% CI | AOR | 95% CI |  | AOR | CI | AOR | CI | AOR | CI | AOR | CI |
| Family Structure |  |  |  |  |  |  |  |  |  |  |  |  |  |
| Two Parents (biological) (Ref) |  |  |  |  |  |  |  |  |  |  |  |  |  |
| Two Parents (One/Both Step/Adoptive) | 2.32 | (0.94, 5.72) | 1.66 | (0.55, 5.02) |  | 0.80 | (0.23, 2.77) | 0.75 | (0.23, 2.50) | 0.27*** | (0.20, 0.37) | 0.28*** | (0.21, 0.38) |
| One Parent | 0.47 | (0.19, 1.15) | 1.03 | (0.59, 1.80) |  | 1.18 | (0.05, 30.57) | 1.07 | (0.04, 27.32) | 1.04 | (0.38, 2.89) | 1.16 | (0.46, 2.96) |
| Other Relatives | 1.01 | (0.56, 1.87) | 0.34* | (0.16, 0.74) |  | 1.12 | (0.27, 4.55) | 0.98 | (0.35, 2.72) | 0.51* | (0.28, 0.90) | 0.43* | (0.20, 0.96) |
| Other Non-Relatives | 3.51** | (1.57, 7.86) | 0.27 | (0.02, 3.04) |  | - |  | - | - | 0.58*** | (0.46, 0.73) | 0.42*** | (0.30, 0.59) |
| Orphan Status (Johannesburg only) |  |  |  |  |  |  |  |  |  |  |  |  |  |
| Not an orphan (Ref) |  |  |  |  |  |  |  |  |  |  |  |  |  |
| Lost only father | - | - | - | - |  | - | - | 2.40* | (1.13, 5.11) | - | - | 0.58** | (0.42, 0.79) |
| Lost only mother | - | - | - | - |  | - | - | 0.50 | (0.06, 3.81) | - | - | 4.96*** | (2.71, 9.08) |
| Lost both parents | - | - | - | - |  | - | - | 1.83 | (0.53, 6.41) | - | - | 4.81** | (1.62, 14.27) |
| Adult Support at Home |  |  |  |  |  |  |  |  |  |  |  |  |  |
| Adult Male Support at Home | 0.96 | (0.92, 1.00) | 1.13** | (1.06, 1.21) |  | 0.97 | (0.88, 1.09) | 0.98 | (0.89, 1.09) | 1.03 |  | 1.04 | (0.90, 1.19) |
| Adult Female Support at Home | 1.06 | (0.82, 1.37) | 0.63*** | (0.52, 0.77) |  | 1.06 | (0.92, 1.23) | 1.07 | (0.91, 1.24) | 0.99 |  | 1.01 | (0.89, 1.15) |
| Home Violence Victimization |  |  |  |  |  |  |  |  |  |  |  |  |  |
| No (Ref) |  |  |  |  |  |  |  |  |  |  |  |  |  |
| Yes | 0.86 | (0.82, 1.37) | 2.65*** | (1.93, 3.63) |  | 1.90 | (0.85, 4.29) | 1.94 | (0.82, 4.57) | 2.18** | (1.32, 3.59) | 2.34** | (1.34, 4.10) |
| * p<0..05, ** p<0.01, *** p<0.001 All analyses are weighted for complex survey design and adjusted for age, school enrollment, unstably housed, and foreign born (Johannesburg only) | | | | | | | | | | | | | |
